# Cognitive Impairement in Non-Cirrhotic Portal Hypertension: Highlights on Physiopathology, Diagnosis and Management

**DOI:** 10.3390/jcm11010101

**Published:** 2021-12-25

**Authors:** Stefania Gioia, Silvia Nardelli, Oliviero Riggio, Jessica Faccioli, Lorenzo Ridola

**Affiliations:** Department of Translational and Precision Medicine, Sapienza University of Rome, 00185 Rome, Italy; nardelli.silvia@gmail.com (S.N.); oliviero.riggio@uniroma1.it (O.R.); jessica.faccioli@uniroma1.it (J.F.); lorenzo.ridola@uniroma1.it (L.R.)

**Keywords:** porto-sinusoidal vascular liver disease, idiopathic non-cirrhotic portal hypertension, portal vein thrombosis, hepatic encephalopathy, porto-systemic shunt

## Abstract

Hepatic encephalopathy (HE) is one of the most frequent complications of cirrhosis. Several studies and case reports have shown that cognitive impairment may also be a tangible complication of portal hypertension secondary to chronic portal vein thrombosis and to porto-sinusoidal vascular disease (PSVD). In these conditions, representing the main causes of non-cirrhotic portal hypertension (NCPH) in the Western world, both overt and minimal/covert HE occurs in a non-neglectable proportion of patients, even lower than in cirrhosis, and it is mainly sustained by the presence of large porto-systemic shunt. In these patients, the liver function is usually preserved or only mildly altered, and the development of porto-systemic shunt is either spontaneous or iatrogenically frequent; HE is an example of type-B HE. To date, in the absence of strong evidence and large cooperative studies, for the diagnosis and the management of HE in NCPH, the same approach used for HE occurring in cirrhosis is applied. The aim of this paper is to provide an overview of type B hepatic encephalopathy, focusing on its pathophysiology, diagnostic tools and management in patients affected by porto-sinusoidal vascular disease and chronic portal vein thrombosis.

## 1. Criteria for the Literature’s Selection

Clinical studies that assessed the prevalence and incidence of any type of hepatic encephalopathy (HE) in patients affected by chronic portal vein thrombosis and porto-sinusoidal vascular disease (PSVD) were included. Studies that evaluated diagnostic tools for the detection of cognitive impairment in this population or that evaluated the efficacy of treatment strategies were included too. No language, publication date, or publication status restrictions were imposed. The studies were identified by searching electronic databases (PubMed and SCOPUS). The last search was run on 28 October 2021. Reference lists of all studies included in the present review were screened for potential additional eligible studies.

One investigator (SG) searched the electronic databases, combining the following keywords: (hepatic encephalopathy AND non-cirrhotic portal hypertension), (hepatic encephalopathy AND porto-sinusoidal vascular disease), (hepatic encephalopathy and portal vein thrombosis), (type B AND hepatic encephalopathy), (hepatic encephalopathy AND idiopathic non-cirrhotic portal hypertension), (hepatic encephalopathy AND nodular regenerative hyperplasia), (cognitive impairment AND non-cirrhotic portal hypertension). Studies were excluded if the title and/or abstract showed that the articles did not meet the selection criteria of our review. For potentially eligible studies, or if the relevance of an article could not be excluded with certitude, we procured the full text. We defined the following exclusion criteria: (1) studies in which HE developed in patients with cirrhosis; (2) studies unrelated to our topic; and (3) studies in which HE developed in patients with a kind of non-cirrhotic portal hypertension other than portal vein thrombosis (PVT) and PSVD. A total of nineteen papers were finally analyzed.

## 2. Definition

Hepatic encephalopathy is a frequent complication and one of the most debilitating manifestations of liver disease, having a relevant impact on the quality of life of the patients and their caregivers [1]. It represents a brain dysfunction caused by liver insufficiency and/or porto-systemic shunting and is characterized by a wide spectrum of neurological or psychiatric abnormalities ranging from subclinical alterations to coma. According to the underlying disease, HE can be divided into: type A, due to acute liver failure, type B, secondary to porto-systemic bypass or shunting, and type C, resulting from cirrhosis [2].

## 3. Historical Point and Pathophysiology

The pathogenesis of hepatic encephalopathy is still much debated and not completely understood. It represents a multifactorial and complex syndrome in which there is an imbalance between production, metabolism and regulation of several neurotoxins and neurotransmitters [3,4] as a result of an interorgan trafficking. According to the most accredited hypothesis, which has its origins as early as 1954 with the studies conducted by Sherlock et al. [5], substances of a predominantly, but not exclusively, nitrogenous nature (ammonium, glutamine, methionine, mercaptans, phenol, indole, serotonin, GABA, etc.) reach the central nervous system, causing the spectrum of symptoms typical of HE. Many studies recognize ammonium as the key pathogenetic element responsible for the astrocytic swelling, known as “astrocyte swelling” [6,7]. In the cirrhotic patient, this process is made possible both by the inability of the liver to catabolize these substances and by the fact that portal hypertension acts as a stimulus for the creation of porto-systemic venous anastomoses. These anastomoses allow “dirty” blood, coming from the intestine/gut, to bypass the liver and reach the brain through systemic circulation, where these toxic substances cause an alteration of neurotransmission. The role of portosystemic bypasses in the development of type C HE is demonstrated by various pieces of evidence: their presence in 46% to 71% of patients with recurrent or persistent HE [8,9], the disappearance or in any case the reduction in the number of HE episodes in these patients after embolization of the shunt [9,10], and finally the development of HE in 25–45% of patients undergoing transjugular intrahepatic portosystemic shunt (TIPS), with evident improvement after revision of the stent [11]. Similar values are also achieved after porto-systemic surgical anastomoses.

In patients with type B HE, by definition, the liver is normally functioning, so the presence of portosystemic shunts would seem to be the main pathogenetic factor. Some animal models of type B HE have been used, in particular in rats, cats, and dogs, and less frequently in rabbits, in which HE was based on the presence of portal-systemic shunting. Moreover, while the presence of portosystemic shunts in humans is a rather rare vascular anomaly, in dogs, it is much more frequent. A 2003 multicenter study [12] found the presence of congenital shunts in 0.18–3.2% of all dogs evaluated, with higher or lower values depending on the breed. Dogs affected by such shunts show clinical symptoms similar to those of human HE. Finally, other studies showed that rats treated with portal-cava anastomosis were sensitive to ammonia administration, which leads to severe encephalopathy [13,14]. The study of these animal models helped to better understand the pathogenesis of type B HE also in humans [15,16].

Most of the works on type B HE conducted on humans come from the Eastern world, and derive from studies on patients with congenital porto-systemic shunts, consequently not related to portal hypertension. Hepatic encephalopathy linked to this type of congenital vascular anomalies was first described by Raskin et al. [17] in 1964, who published the clinical case of a patient with HE associated with a large spontaneous intrahepatic shunt. In the following years, the interest of the scientific community towards this clinical condition increased, above all due to the development of diagnostic techniques for non-invasive images, through which it was possible to obtain increasingly accurate images of the portal venous system (Doppler Ultrasound, contrast enhanced CT-scan and MRI). The Japanese Society for the Study of Liver Disease is the organization most interested in bypass HE. Thanks to the results of a national survey, in 2000, Watanabe published a review specifically focused on the subject [18]. From this investigation, it emerged that many patients with shunt-related HE were wrongly diagnosed as being affected by dementia, psychiatric or neurological disorders, or even by cirrhosis or acute liver failure, and therefore were submitted to prolonged hospitalizations and inappropriate medical interventions. Hence the importance, according to Watanabe, of searching for the presence of spontaneous porto-systemic shunts in all patients with typical symptoms and signs of HE even in the absence of an altered liver function. Watanabe identified both congenital vascular anomalies (patent ductus venous duct, absence of portal vein, arteriovenous malformations, rupture of intrahepatic portal varices, Rendu-Osler-Weber disease, etc.) and acquired (post abdominal surgery, trauma, liver biopsy, etc.) as causes of the formation of these collateral vessels. However, since, in most cases, it was not possible to find a specific cause, the author hypothesized that these shunts were due to portal hypertension, which disappeared after the development of the aforementioned anastomoses due to their decompressive action.

Patients affected by non-cirrhotic portal hypertension (NCPH) theoretically represent an ideal model in which to study type B HE, as they maintain preserved liver function for a long time, but have portal hypertension, which is an important stimulus to shunt formation (spontaneous acquired porto-systemic). Therefore, in this review, we focused on the prevalence, the diagnosis and management of HE occurring in patients affected by idiopathic non-cirrhotic portal hypertension (INCPH), recently named as porto-sinusoidal vascular disease (PSVD), and chronic portal vein thrombosis (PVT), which represent the most frequent vascular liver diseases causing NCPH in the Western world [19,20,21,22,23,24]. In the presence of portal hypertension, the porto-systemic shunts develop both passively, following the reopening of collapsed embryonic vessels and the inversion of flow in pre-existing vessels (in fact, physiologically there are numerous portosystemic anastomoses), and actively thanks to an increase in VEGF levels [25,26].

Moreover, Das et al. showed that patients with PSVD had cerebral alterations typically observed in cirrhotic patients [27]. In greater detail, they confirmed that the majority of cirrhotics have a hyperintense globus pallidus on T1 W MRI images, and they showed that none of the patients with PVT but more than half of the patients with PSVD had similar radiological findings. The diagnosis of PSVD or cirrhosis was the only independent predictor of the presence of these findings. That cerebral alteration has been attributed to the effects of manganese, which is deposited in excess in the brain of cirrhotics [28,29,30,31,32], and that normally, it is mainly cleared by the liver and excreted in bile [33], and its deposition in cirrhotics is probably due to lower biliary clearance secondary to the hepatocellular damage and to porto-systemic bypass. In cirrhosis, both mechanisms are involved, and this makes it hard to define what is responsible for these alterations. The authors speculate that, as with cirrhotics, but unlike PVT, patients with PSVD have an increased fasting arterial ammonia and abnormal ammonia tolerance test [34], making them prompt in developing HE under appropriate stress. Finally, the finding that the studied cerebral changes were not observed in patients with PVT is in opposition to previous results [35].

## 4. Prevalence of HE

The literature on hepatic encephalopathy in patients with portal hypertension due to portal vein thrombosis is mostly based on studies conducted in the Eastern world [36,37,38,39], where this clinical condition is more frequent, or in pediatric populations [40,41,42]. The main results of these studies are summarized in Table 1. Sharma et al. [36] showed that the prevalence of minimal hepatic encephalopathy (MHE) assessed by psychometric tests and critical flicker frequency (CFF) was 35.5% in patients with chronic extrahepatic portal vein obstruction (EHPVO). The same group demonstrated [37], in a cohort of 32 patients with EHPVO followed up for 1 year, that 12 patients were affected by MHE at baseline, that 75% of them continued to have MHE at follow-up, and that one of the patients without MHE developed it later. In the short time of follow-up, none of the patients developed overt HE. The presence of MHE in these patients was strongly associated with a higher expression of ammonia, pro-inflammatory cytokines, and brain glutamine levels.

The literature on patients with PSVD is based on several individual case reports [48,49] and some studies, principally conducted in the Western world, describing HE as a tangible complication of the disease, mainly related to the presence of large porto-systemic shunts (Table 1) [43,44,45].

Evaluating the prevalence of hepatic encephalopathy (minimal and overt) in 51 patients affected by NCPH in comparison with that of a control group of cirrhotic patients [44], Nicoletti et al. showed that, even lower than that observed in cirrhotic patients, a cognitive impairment was detectable in a relevant proportion of patients with non-cirrhotic portal hypertension, with no difference between the patients with chronic portal vein thrombosis and PSVD. The presence of a large portal-systemic shunt (spontaneous or iatrogenic) was considered the main risk factor for HE in these patients, as it was identified in 71% of the patients with cognitive impairment. Another study showed that in patients affected by NCPH, the incidence of OHE was similar, while the prevalence of MHE was lower than that of cirrhosis patients. The authors confirm that together with upper gastrointestinal bleeding and infection, a portosystemic shunt was an independent factor for HE [46].

Additionally, post-TIPS HE is a not infrequent complication of portal hypertension due to PSVD [47]. In a European cohort of 41 patients affected by PSVD and submitted to TIPS as the treatment of portal hypertension-related complications [45], HE was an in-hospital complication of two patients, while at long-term follow-up, overt HE occurred in 31% of the patients, and the one-year rate of overt HE was 24%. In two patients, HE was severe enough to require shunt reduction [45].

## 5. Diagnosis

The tests and the methods used to diagnose hepatic encephalopathy in patients with NCPH are the same currently used for the diagnosis of HE in cirrhotic patients.

### 5.1. Diagnosis of Overt Hepatic Encephalopathy

The diagnosis of overt HE is mainly based on clinical examination. Some scales are used to stage the severity of the encephalopathy, and with this aim, the most applied is the West Haven scale, which still represents the gold standard. The diagnosis of cognitive dysfunctions is not difficult; less easy is the attribution of them to overt HE, and that is why the exclusion of other causes of mental alteration by laboratory and radiological assessment is often required [2,50].

### 5.2. Diagnosis of Covert Hepatic Encephalopathy

For the diagnosis of minimal/covert HE, the same tools used for cirrhotic patients are used in NCPH patients, and they include paper-pencil tests (the psychometric hepatic encephalopathy score- PHES), computerized tests such as continuous reaction time, the inhibitory control test, the SCAN test and the Stroop test, or neurophysiological tests including the CFF and EEG. Clinicians may use tests for the diagnosis of MHE with which they are familiar that have been validated for use in this patient population [2,50,51,52,53].

In the studies exploring the prevalence of minimal HE in patients affected by EHPVO, especially in children, the most used tools for the assessment of HE were psychometric tests and CFF. In the study by Yadav, the superiority of psychometric tests in comparison to CFF was demonstrated. The same observation resulted from a study by Srivastava [42]. In a recent study by Suresh et al., the diagnostic accuracy of the computerized Stroop test for the assessment of MHE in an Indian pediatric cohort of patients with EHPVO was investigated in comparison to other validated tests. The authors observed that the Stroop test can be useful to detect MHE in children and identify a subgroup of patients to be submitted to psychometric tests in clinical care. Nicoletti et al., as previously reported, used two categories of tests to evaluate the presence of minimal/covert HE [2]: the PHES and the Scan battery. The accuracy of the Scan battery and of PHES in the detection of MHE was similar, but some discordance was observed, suggesting that the two tests have different levels of difficulty [54] (the scan test is more complex than PHES), and that they explore different domains of cognitive function.

## 6. Treatment

To date, the treatment of overt hepatic encephalopathy occurring in patients affected by NCPH is the same as that of HE in cirrhotic patients.

The initial management includes a prompt start of care of hospitalized patients with HE, including the identification and the treatment of co-existing causes; the identification and correction of precipitating factors and the start of empirical treatment targeted the reduction in ammonia levels. The cornerstones of medical treatment of overt HE include nonabsorbable disaccharides, such as lactulose, and antibiotics, such as rifaximin, where lactulose is recommended for the prevention of recurrent episodes of HE after the initial episode, and rifaximin as an add-on to lactulose is recommended for the prevention of recurrent episodes of HE after the second episode. Alternative therapies, such as oral branched-chain amino acids, intravenous L-ornithine L-aspartate, and probiotics have been studied and used in cirrhotic patients, but no data in patients with NCPH have been provided [2,53]. Whether applying in patients with NCPH the same therapeutic strategies used in cirrhotic patients is correct is unknown. Although HE is a less frequent complication of a less frequent disease, more cooperative studies are needed to identify the best approach to treat hepatic encephalopathy in patients with NCPH.

**Figure 1:**
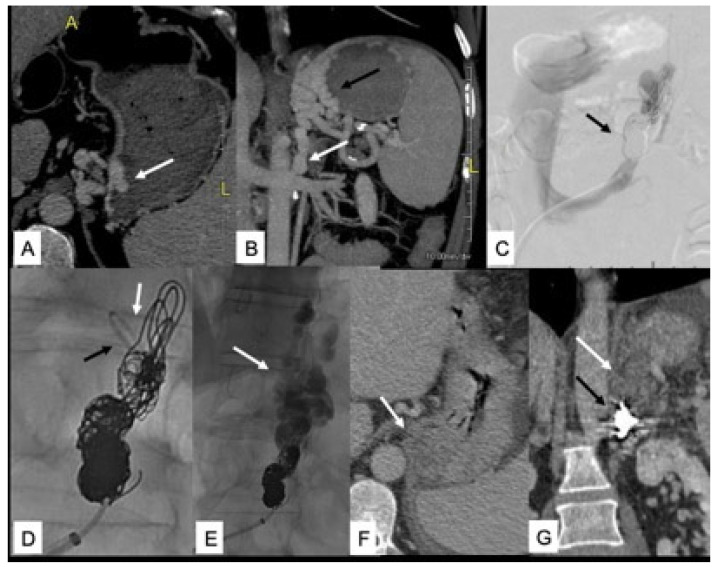
(**A**,**B**) Pre-procedural CT axial and coronal reformats show intra- and extramural gastric varices (GV) (white arrow in (**A**) and black arrow in (**B**)) and GRS (white arrow in (**B**,**C**)). Coil embolization of GRS (black arrow) with persistent shunt patency. (**D**) Angiographic catheter distal to coils (black arrow) and coaxial microcatheter looped backward for NBCA injection (white arrow). (**E**) Extensive filling of GV with gelfoam and contrast media (white arrow). (**F**,**G**) Post-procedural CT shows complete thrombosis of GV and GRS (white arrow) and coils in the GRS (black arrow).

As hepatic encephalopathy occurring in patients affected by portal vein thrombosis or idiopathic non cirrhotic portal hypertension is a type B HE, mainly sustained by the presence of large porto-systemic shunts, the radiological occlusion of the shunt may represent a fundamental approach in patients with persistent HE, despite an adequate medical treatment. [43,55] Radiological techniques such as plug-assisted retrograde transvenous obliteration (PARTO) or coil-assisted retrograde transvenous obliteration (CARTO) are currently used to treat recurrent or persistent HE [56,57,58], as well as gastric varices often present in these patients (Figure 1). Finally, in patients with persistent post-TIPS HE, the reduction in the caliber of the stent or its occlusion must be evaluated.

Finally, as in cirrhosis, the necessity to treat MHE is still debated. Despite its clinical implications (impairment, poor quality of life, etc.), guidelines state that the treatment of minimal/covert HE in cirrhotic patients is to be evaluated on a case-by-case basis [53,59,60].

However, some studies observed an improvement in psychometric tests in the majority of the EHPVO pediatric patients with MHE after therapy with lactulose, and that such treatment was well-tolerated [61]. These results confirm a previous study by Sharma were lactulose seemed to be effective in the treatment of minimal hepatic encephalopathy in patients with portal vein thrombosis, and that patients with cognitive impairment and with porto-systemical shunts had a better response to lactulose than the patients without any collaterals [38]. In the patients who responded to lactulose, the blood ammonia levels significantly reduced, while in the patients who were non-responders to the treatment, they did not.

## 7. Conclusions and Future Directions

Type B HE can be considered a complex and multidimensional cognitive deficit that is not infrequently found in patients with NCPH and that shares substantial physio-pathological bases with type C HE. Both the presence of shunts per se and the neurotoxic effect of toxins of intestinal origin play a fundamental role in determining and supporting alterations in mental status, even in the absence of hepatocellular damage. Moreover, a multidisciplinary approach for the best management of these patients is often needed. In fact, patients affected by non-cirrhotic portal hypertension may develop all the sequelae of portal hypertension, such as portal hypertensive bleeding or refractory ascites often requiring TIPS placement. However, the development of HE episodes that are also poorly responsive to medical treatment after this procedure, which could require stent revision with the reduction in its caliber or occlusion, is not rare.

Finally, a thorough understanding of the impact of HE in NCPH patients cannot fail to consider the need for reliable epidemiological data. Therefore, further studies will also be needed to establish the exact prevalence and incidence of cognitive impairments in these patients. In fact, only a thorough knowledge of all the facets of the problem will allow us to promptly identify patients at risk, study the cognitive deficit extensively and undertake appropriate therapies.

## Figures and Tables

**Table 1:** Published studies on hepatic encephalopathy in patients with non-cirrhotic portal hypertension (NCPH): chronic portal vein thrombosis (PVY) or porto-sinusoidal vascular disease (PSVD).

Author, Year	N° of Patients	Type of NCPH	Prevalence of HE	Type of HE
Sharma et al., 2008 [36]	34	PVT	37.3%	MHE
Sharma et al., 2012 [38]	70	PVT	43%	MHE
Srivastava et al., 2011 [39]	20	PVT	60%	MHE
Yadav et al., 2010 [40]	22	PVT	32%	MHE
D’Antiga et al., 2014 [41]	13	PVT	45%	MHE
Srivastava et al., 2010 [42]	42	PVT	36%	MHE
Siramolpiwat et al., 2014 [43]	84	PSVD	7%	OHE
Nicoletti et al., 2016 [44]	51	PVT and PSVD	34% (PVT)/25% (PSVD) 5.7% (PVT)/12.5% (PSVD)	MHE OHE
Bissonnette et al., 2016 [45]	41	PSVD	31%	OHE
Liu et al., 2019 [46]	150	PSVD	4.7% 32.7%	OHE MHE
Lv et al., 2019 [47]	76	PSVD	16%	OHE

## Data Availability

Data sharing is not applicable to this article as no new data were created or analyzed in this study.

## Abbreviations

HE: hepatic encephalopathy; GABA: gamma-aminobutyric acid; TIPS: transjugular intrahepatic portosystemic shunt; PSVD: porto-sinusoidal vascular liver disease; PVT: portal vein thrombosis; NCPH: non-cirrhotic portal hypertension; INCPH: idiopathic non-cirrhotic portal hypertension; EHPVO: extrahepatic portal vein obstruction; CT: computed tomography; MRI: magnetic resonance imaging; VEGF: vascular-endothelial growth factor; CRT: continuous reaction time; ICT: inhibitory control test; CFF: critical flicker frequency; EEG: electroencephalography; CARTO: coil-assisted retrograde transvenous obliteration; PARTO: plug-assisted retrograde transvenous obliteration.
